# Id2 deletion attenuates *Apc*-deficient ileal tumor formation

**DOI:** 10.1242/bio.012252

**Published:** 2015-07-10

**Authors:** Kyoko Biyajima, Fumihiko Kakizaki, Xiaodong Shen, Kentaro Mori, Manabu Sugai, M. Mark Taketo, Yoshifumi Yokota

**Affiliations:** 1 Division of Molecular Genetics, Department of Biochemistry and Bioinformative Sciences, School of Medicine, Faculty of Medical Sciences, University of Fukui, 23-3 Matsuoka-Shimoaizuki, Eiheiji-cho, Yoshida-gun, Fukui 910-1193, Japan; 2 Department of Pharmacology, Graduate School of Medicine, Kyoto University, Yoshida Konoé-cho, Sakyo-ku, Kyoto 606-8501, Japan

**Keywords:** Apc, c-Myc, Id2, Mxd1, Ileal tumor initiation

## Abstract

The expression level of inhibitor of DNA binding 2 (Id2) is increased in colorectal carcinomas and is positively correlated with poor prognosis. However, the functional significance of Id2 in intestinal tumorigenesis has not been fully defined using genetic approaches. Here, we show that Id2 promotes ileal tumor initiation in *Apc*-deficient mice. Expression of Id2 was stimulated by Wnt signaling through the enhancer region of the *Id2* promoter at the early stage of tumorigenesis in *Apc*^+/Δ716^ (*Apc*^Δ716^) mice. Genetic depletion of *Id2* in *Apc*^Δ716^ mice caused ∼80% reduction in the number of ileal polyps, but had little effect on tumor size. Notably, the lack of Id2 increased the number of apoptotic cells in the normal crypt epithelium of the mice. Furthermore, DNA microarray analysis revealed that the expression level of Max dimerization protein 1 (Mxd1), known as a c-Myc antagonist, was specifically increased by Id2 deletion in the ileal intestinal epithelium of *Apc*^Δ716^ mice. In contrast, the protein level of c-Myc, but not the mRNA level, was decreased by loss of Id2 in these mice. These results indicate that loss of Id2 inhibits tumor initiation by up-regulation of Mxd1 and down-regulation of c-Myc in *Apc*^Δ716^ mice.

## INTRODUCTION

Somatic mutation of the adenomatous polyposis coli (*APC*) tumor suppressor gene occurs in most colorectal cancers, and is an early event in the development of sporadic colorectal cancer (Powell et al., 1992). A germ-line mutation of *APC* is also responsible for the genetic disorder familial adenomatous polyposis (FAP) (Kinzler and Vogelstein, 1996; Taketo and Edelmann, 2009). Mutation of *APC* leads to activation of Wnt signaling via β-catenin stabilization. The accumulated β-catenin then translocates to the nucleus where it stimulates the expression of Wnt target genes through interaction with T-cell factor/lymphocyte enhancer factor (TCF/LEF) transcription factors (Fodde et al., 2001).

Id2, a member of the Id family, has a helix-loop-helix (HLH) motif but lacks a DNA-binding domain. Therefore, Id2 proteins prevent bHLH proteins from interacting with DNA in a dominant negative manner (Ruzinova and Benezra, 2003; Yokota and Mori, 2002). Since bHLH transcription factors play an important role in multiple processes of cell differentiation and proliferation, Id2 is also involved in these processes (Ruzinova and Benezra, 2003; Yokota, 2001). The expression level of Id2 is generally low in normal adult tissues. However increased expression of Id2 is observed in various human cancers including colorectal cancer, and often correlates with poor prognosis (Gray et al., 2008; Perk et al., 2005; Rockman et al., 2001; Wilson et al., 2001). Interestingly, previous studies have shown that transcription from the *ID2* promoter is increased by overexpression of β-catenin (Rockman et al., 2001). Electrophoretic mobility shift assays have also revealed that the *ID2* promoter contains a functional TCF-4 binding site (Rockman et al., 2001). Furthermore, knockdown of Id2 expression not only inhibits proliferation but also induces apoptosis in colorectal cancer cells that have mutations in components of the Wnt signaling pathway (Gray et al., 2008). On the other hand, overexpression of Id2 increases anchorage-independent survival of colon cancer cells (Rockman et al., 2001). These circumstantial evidences suggest that Id2 is likely to be important for colorectal tumorigenesis *in vivo*. However, the functional significance of Id2 in intestinal tumorigenesis has not been fully defined using genetic approaches.

To elucidate the physiological role of Id2 in intestinal tumorigenesis using a genetic approach, we introduced an Id2 null mutation into *Apc*^Δ716^ mice, a model for human FAP (Oshima et al., 1995a; Yokota et al., 1999). We have demonstrated here that Id2 plays an essential role in ileal tumor initiation in *Apc*^Δ716^ mice.

## RESULTS

### Expression of Id2 is stimulated at early stages of polyp formation in *Apc*^Δ716^ mice

**Fig. 1.**
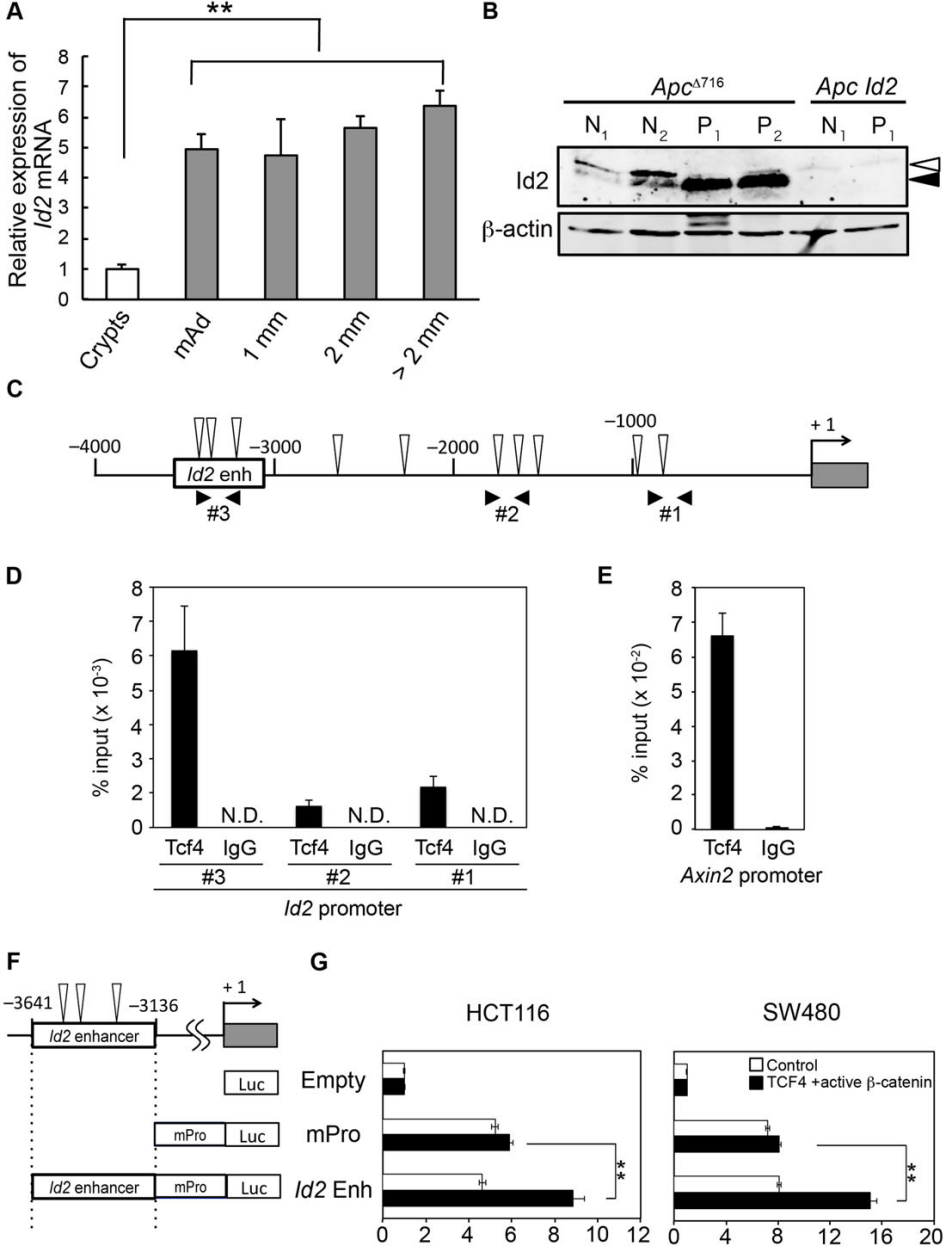
**Expression of Id2 is increased in the intestinal tumor epithelial cells of *Apc*^Δ716^ mice.** (A) Expression level of *Id2* mRNA determined by qRT-PCR. Epithelial cells were collected by laser-microdissection from intestinal polyps and adjacent normal crypts (crypt epithelial cells) of *Apc*^Δ716^ mice. Microadenomas (mAd) and adenomas 1 mm, 2 mm and >2 mm in diameter were analyzed. The results are given as means±s.d. (*n*=3). **P<0.01. (B) Expression level of the Id2 protein in intestinal polyps and adjacent normal tissues of *Apc*^Δ716^ and *Apc Id2* mice was analyzed by western blotting. *N*, normal tissue; *P*, polyp. Open arrowhead, nonspecific band; closed arrowhead, endogenous Id2. β-actin, loading control. (C) Schematic representation of the locus of the *Id2* enhancer region (*Id2* enh) in the *Id2* promoter. The gray rectangle indicates the first exon of *Id2*. The numbers indicate the distance from the *Id2* transcriptional start site. The open arrowheads indicate Tcf/Lef binding consensus sequences. (D,E) ChIP assays. Chromatin from small intestinal polyps of *Apc*^Δ716^ mice was immunoprecipitated with antibodies against Tcf4 or IgG as a control, followed by qPCR using (D) primer pairs (#1–#3) spanning the mouse *Id2* promoter locus shown in C or (E) primers specific for the *Axin2* promoter. The results are presented as percentage immunoprecipitated over input and are representative of three independent experiments. N.D., not detected. The results are given as means±s.d. (F) Schematic representation of the luciferase (Luc) expression constructs containing the *Id2* enhancer with/without a CMV-minimal promoter (mPro). (G) Luciferase reporter gene assays for active β-catenin mediated activation of the CMV-mPro. HCT116 (left) or SW480 (right) cells were transfected with a reporter construct and an expression vector for active β-catenin and TCF4. Bars indicate relative luciferase activities calibrated to those of the promoter-less luciferase construct. Error bars show standard deviation of triplicate samples. Empty, promoter-less luciferase construct; mPro, luciferase construct containing only CMV minimal promoter; *Id2* Enh, luciferase construct containing the CMV minimal promoter and *Id2* DNA fragment of −3641 to −3136. **P<0.01.

To investigate the potential role of Id2 in intestinal tumorigenesis, we first determined the mRNA levels of *Id2* in the tumor epithelium of *Apc*^Δ716^ mice by qRT-PCR analysis. The tumor and adjacent normal epithelia were collected by laser microdissection. As shown in Fig. 1A, the level of *Id2* in the microadenoma epithelium (≤0.5 mm in diameter) was 5 times higher than that in normal crypts in *Apc*^Δ716^ mice. We also found that tumor epithelial cells from adenomas larger than 0.5 mm in diameter showed essentially the same level of *Id2* mRNA as that in microadenomas (Fig. 1A). Consistent with the qRT-PCR data, the levels of Id2 protein in *Apc*^Δ716^ adenomas were 3 times higher than that in the adjacent normal intestinal tissues (Fig. 1B). It has been reported that *ID2* is a downstream target gene of Wnt signaling in human colon cancer cell lines (Rockman et al., 2001). To determine if mouse *Id2* is also a target gene of Wnt signaling, we performed ChIP-PCR analysis of the binding of Tcf4 to the promoter region of the *Id2* gene. Sequencing analysis revealed ten putative Tcf/Lef-binding consensus sequences in the *Id2* promoter region spanning from −4000 to −1 (Fig. 1C; supplementary material Fig. S1). As expected, Tcf4 bound to the DNA region spanning from −3300 to −3146 of the *Id2* distal promoter (Fig. 1D). We also confirmed the binding of Tcf4 to the promoter region of the known β-catenin target gene *Axin2* (Fig. 1E). To test this Tcf4 binding region for enhancer activity, we conducted luciferase reporter assays using the constructs shown in Fig. 1F. The putative *Id2-*enhancer fragment significantly activated Wnt-signaling dependent transcription from the CMV minimal promoter in two colorectal cancer cell lines (HCT116 and SW480) (Fig. 1G). Therefore, these results indicate that Id2 expression is stimulated by Wnt signaling through the enhancer region, in tumor epithelial cells of *Apc*^Δ716^ mice at an early stage of tumorigenesis.

### Loss of Id2 inhibits ileal tumor initiation of *Apc*^Δ716^ mice

**Fig. 2.**
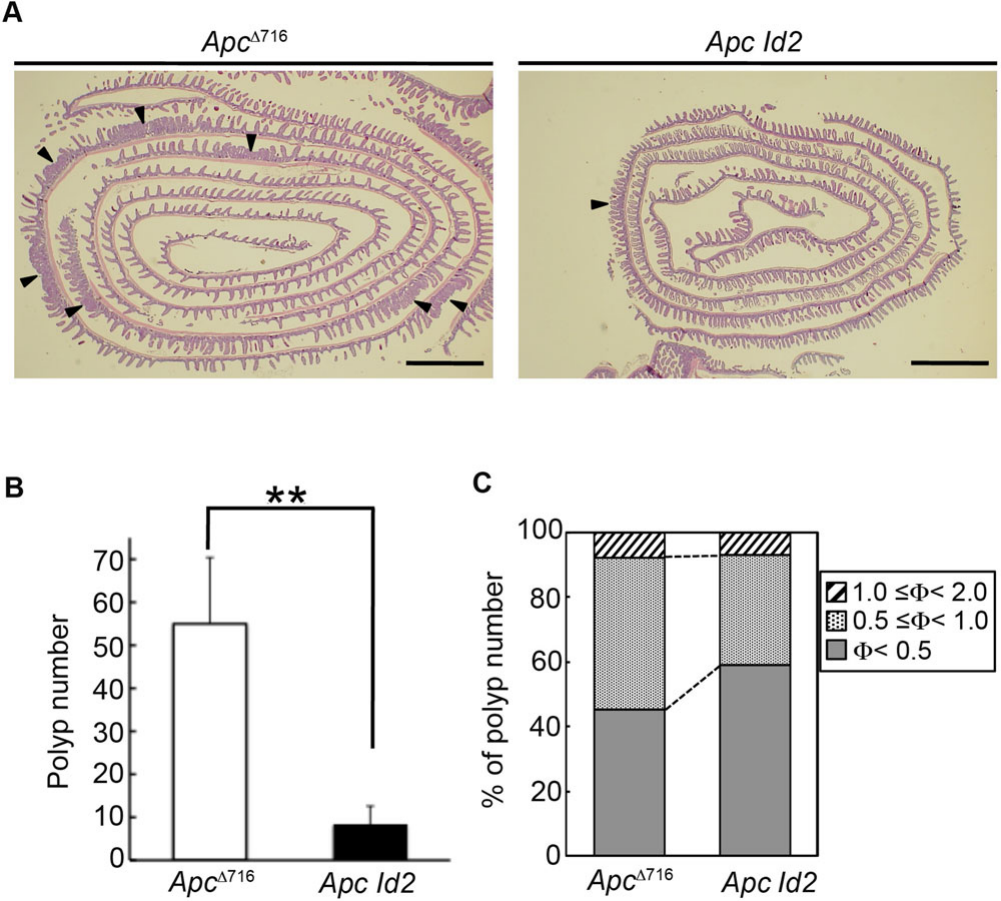
**Loss of Id2 attenuates ileal tumor formation in *Apc*^Δ716^ mice.** (A) Representative Swiss rolls of the ileum from *Apc*^Δ716^ and *Apc Id2* mice stained with H&E. Arrowheads indicate polyps. Scale bars, 1 mm. (B) Total number of polyps in the *Apc*^Δ716^ and *Apc Id2* mice at 16 weeks of age as assessed using a dissecting microscope. The results shown are the mean number of polyps/mouse±s.d. (*n*=7–8). **P<0.01. (C) Size distribution of the polyps. Polyps were classified according to their diameter in millimeters. The results are shown as percentages for the respective size classes (*n*=7–8). Φ indicates polyp diameter.

To determine the role of increased Id2 expression in the tumorigenesis of *Apc*-deficient intestinal adenomas, we crossed the *Apc*^Δ716^ mice with *Id2^−^*^/*−*^ mice twice to generate *Apc*^+/Δ716^ *Id2^−^*^/*−*^ (*Apc Id2*) mice. Because *Id2^−^*^/*−*^ mice are perinatal lethal on a C57BL/6 background (Ghosh et al., 2014), the *Apc*^Δ716^ allele on the C57BL/6 background was transferred to the 129/Sv genetic background. As shown in Fig. 1B, expression of Id2 protein could not be detected in the normal intestinal tissues or intestinal polyps of the *Apc Id2* mice. Due to intestinal distortion between the jejunum and ileum caused by loss of Id2 (Russell et al., 2004), the *Apc Id2* mice became moribund around 16 weeks of age. We therefore euthanized these mutant mice at this age. Since the *Apc*^Δ716^ mice in the 129/Sv genetic background formed few polyps in the colon at this age, we therefore focused on small intestinal polyps in these mice. Notably, loss of Id2 resulted in a markedly decreased ileal polyp number in *Apc*^Δ716^ mice (Fig. 2A,B). Specifically, the *Apc*^Δ716^ mice developed 55±15 ileal polyps at the age of 16 weeks. On the other hand, in *Apc Id2* mutant mice of the same age, the total ileal polyp number was significantly lower (8±4 polyps), showing an 80% reduction in the number found in the *Apc*^Δ716^ mice (*P*=0.0001 Fig. 2B). Interestingly, there was essentially no difference in the number of tumors in the duodenum or the jejunum between the two mutant mice (supplementary material Fig. S2A,B). To further evaluate the effect of the loss of Id2 in the early stage of tumorigenesis in the *Apc*^Δ716^ mice, we compared the number of microadenomas between the *Apc*^Δ716^ and *Apc Id2* mice at 4 weeks of age, before any macroscopically visible tumors could be observed. As shown in supplementary material Fig. S2C, the *Apc*^Δ716^ mice had formed 4±0.9 polyps in the ileum by 4 weeks of age. In contrast, the number of polyps in the littermate *Apc Id2* mice was 25% that of the *Apc*^Δ716^ mice of the same age (1±0.6 polyps, *P*=0.00009. supplementary material Fig. S2C). Since knockdown of Id2 was reported to suppress the proliferation of colon cancer cells (Gray et al., 2008), we next determined polyp size distribution in these mice. We found that the percentage of ileal polyps with a diameter of 0.5–1 mm in the *Apc Id2* mice was lower than that in the *Apc*^Δ716^ mice (*Apc*^Δ716^, 47% versus *Apc Id2*, 34%; *P*=0.02; Fig. 2C). Furthermore, the percentage of polyps with a diameter of less than 0.5 mm in the *Apc Id2* mice was higher than in the *Apc*^Δ716^ mice (*Apc*^Δ716^, 45% versus *Apc Id2*, 59%; *P*=0.02; Fig. 2C). However, there was no difference in the percentage of large (1−2 mm) ileal polyps between the two mutant mice (Fig. 2C). These results indicate that Id2 deficiency has a much smaller effect on tumor size than it has on tumor number and that Id2 plays an important role in ileal tumor initiation in *Apc*^Δ716^ mice.

It has been reported that *Id2^−/−^* mice also form intestinal polyps (Russell et al., 2004). Because it is known that loss of heterozygosity (LOH) of *Apc* stabilizes β-catenin and promotes its nuclear accumulation, nuclear β-catenin expression was observed in Apc loss-induced epithelial adenoma (supplementary material Fig. S3C). However, nuclear β-catenin accumulation was not detected in Id2 loss-induced epithelial tumor cells (supplementary material Fig. S3F). Because of these differences, Apc-deficient adenomas can be distinguished from Id2 loss-induced polyps in *Apc Id2* mice. β-catenin staining revealed that the percentage of Apc-deficient adenomas was ∼96% in *Apc Id2* mice (supplementary material Table S1). This result suggests that the tumors derived from Id2 loss are negligible in number in this analysis.

### The Id2 protein is specifically expressed in the intestinal crypt epithelium

**Fig. 3.**
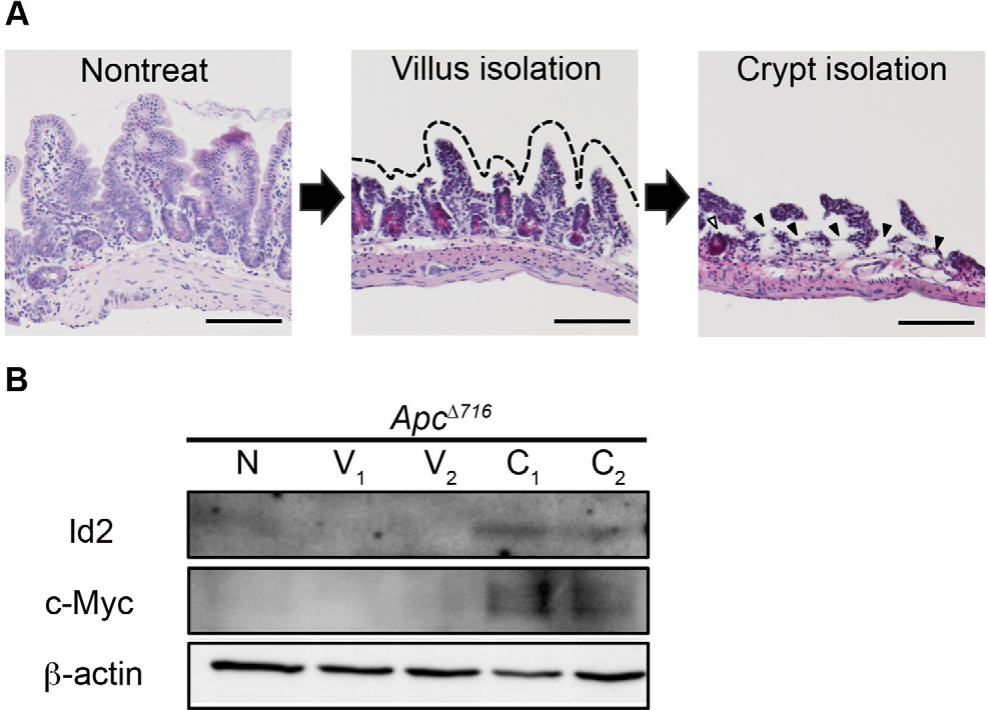
**The Id2 protein is specifically expressed in the intestinal crypt epithelium.** (A) H&E staining of normal small intestinal tissues of *Apc*^Δ716^ mice before (left panel) and after EDTA treatment to remove villi (middle panel) and crypts (right panel). The dashed line indicates the areas from which the villi were removed. Closed arrowheads indicate the areas from which the crypt epithelium was removed. An open arrowhead indicates the remaining crypt area. Scale bars, 100 µm. (B) Expression of the Id2 protein analyzed by western blotting. Protein samples were isolated from the normal epithelium of villi and crypts of *Apc*^Δ716^ mice, as shown in A. To confirm that the isolated cells were of crypt cell origin, each sample was also subjected to western blotting of the c-Myc protein. N, normal tissue; V, villus epithelium; C, crypt epithelium. β-actin, loading control.

It has been reported that polyp formation is initiated by LOH at the *Apc* locus in the proliferative zone cells of the intestinal crypts of *Apc*^Δ716^ mice (Oshima et al., 1995a). To determine if Id2 is expressed in intestinal crypts, we assessed its protein expression level along the crypt-villus axis of the intestinal epithelium in these mice. We first separated the normal villus and crypt epithelium from the surrounding tissue. H&E staining showed that villi and crypts were successfully isolated (Fig. 3A). Western blotting showed prominent Id2 expression in the crypt epithelium but not in the villus epithelium within the normal epithelial compartment of the *Apc*^Δ716^ mice (Fig. 3B).

Because the loss of Id2 specifically suppresses ileal tumor initiation in *Apc*^Δ716^ mice, we confirmed the expression patterns of Id2 and other Id-family members (Id1 and Id3) along the proximal-distal axis of the small intestine. The expression levels of Id1, Id2, and Id3 remained unchanged throughout the small intestine, although the levels of Id1 and Id3 were increased by Id2 deletion (supplementary material Fig. S4A-C). These results indicate that the ileal specific phenotype is not attributable to different expression patterns among Id-family members along the proximal-distal axis of the small intestine.

It is known that an additional mutation in the *Cdx2* gene in *Apc*^Δ716^ mice reverses polyp localization, shifting most polyps to the colon (Taketo and Edelmann, 2009). It has also been reported that a conditional mutation in the *Gata4* gene in the jejunum of adult mice results in a partial jejunal-to-ileal transformation (Bosse et al., 2006). We therefore evaluated the expression of the *Gata4, Cdx1*, and *Cdx2* genes. However, the expression levels of *Gata4*, *Cdx1*, and *Cdx2* were not altered by the loss of Id2 (supplementary material Fig. S5). These results suggest that Id2 promotes ileal tumor initiation in the proliferating crypt epithelium of *Apc*^Δ716^ mice without the effect on the location of the polyps.

### Id2 promotes ileal tumor initiation through inhibition of apoptosis in intestinal crypts of *Apc*^Δ716^ mice

**Fig. 4.**
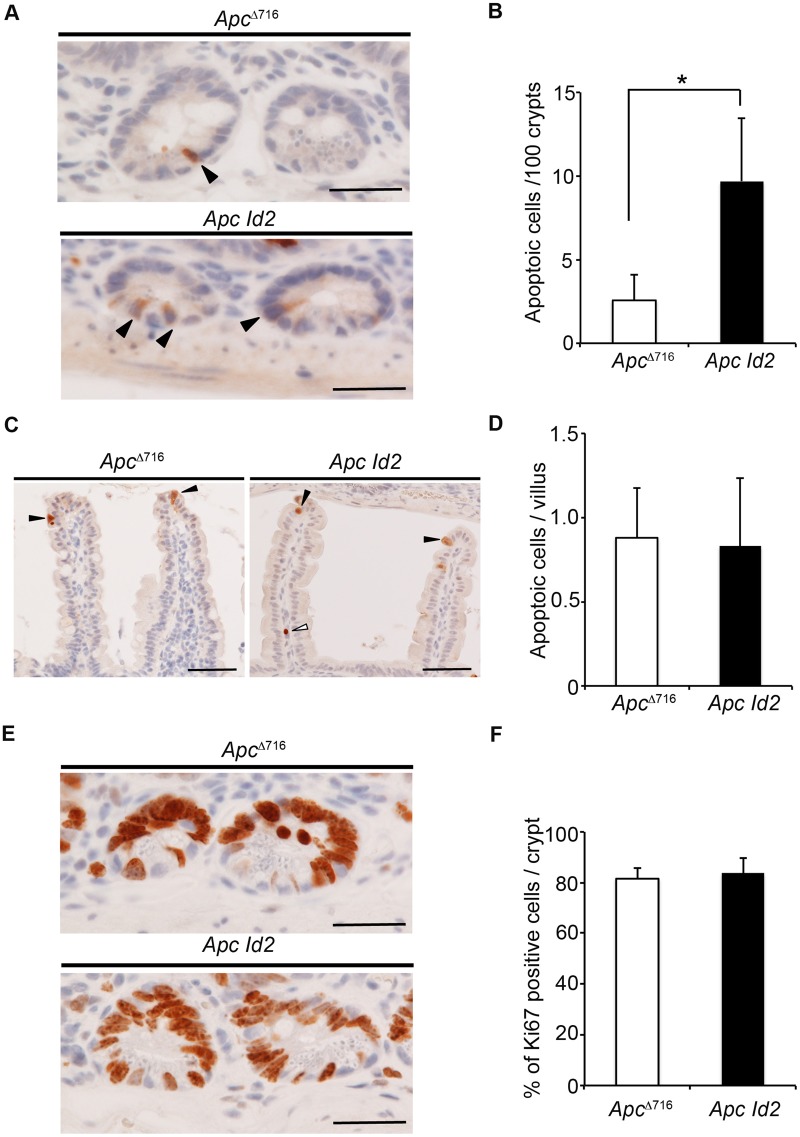
**Loss of Id2 increased apoptosis in the intestinal crypt of *Apc*^Δ716^ mice.** (A-D) Apoptotic cells (closed arrowheads) in (A) the ileal crypts and (C) villi of representative *Apc*^Δ716^ and *Apc Id2* mice, visualized by immunohistochemical analysis using an antibody against cleaved caspase-3. Closed arrowheads, cleaved caspase-3 positive epithelial cells; open arrowhead, cleaved caspase-3 positive stromal cell. Scale bars, 25 µm and 50 µm (A and C, respectively). Quantification of apoptotic cells in the (B) ileal crypts and (D) villi of *Apc*^Δ716^ and *Apc Id2* mice. (E) Cell proliferation in the ileal crypts of representative *Apc*^Δ716^ and *Apc Id2* mice, visualized by immunohistochemical analysis using an anti-Ki67 antibody. Scale bars, 25 µm. (F) Quantification of Ki67-positive cells in the ileal crypts of *Apc*^Δ716^ and *Apc Id2* mice. The results are given as means±s.d. (*n*=3). *P<0.05.

To analyze the mechanism of Id2-mediated tumor initiation, we next investigated the effect of Id2-deficiency on the proliferation and apoptosis of normal crypt cells of *Apc*^Δ716^ mice. Immunohistochemistry of cleaved caspase-3, which is a marker of apoptosis, revealed that the rate of apoptotic cells per 100 crypts in *Apc Id2* mice was 3.7 times higher than that in *Apc*^Δ716^ mice. (*Apc*^Δ716^, 2.6±1.5 versus *Apc:Id2*, 9.7±3.8; *P*=0.04; Fig. 4A,B). On the other hand, loss of Id2 did not affect apoptosis of villus epithelial cells in *Apc*^Δ716^ mice (Fig. 4C,D). Furthermore, there was no difference in the percentage of Ki67 positive cells in normal crypt epithelium between *Apc*^Δ716^ and *Apc Id2* mice. (*Apc*^Δ716^, 82±4.1 versus *Apc:Id2*, 84±5.7; *P*=0.15; Fig. 4E,F). In addition, the absence of Id2 in ileal tumor epithelium did not alter the proliferative capacity or the apoptosis rate in *Apc*^Δ716^ mice (supplementary material Fig. S6). These data suggest that Id2 promotes tumor initiation with inhibition of apoptosis in the intestinal crypt cells of *Apc Id2* mice.

### Loss of Id2 reduces the protein level of c-Myc and increases the mRNA level of its antagonist Mxd1 in ileal crypt epithelium of *Apc*^Δ716^ mice

**Fig. 5.**
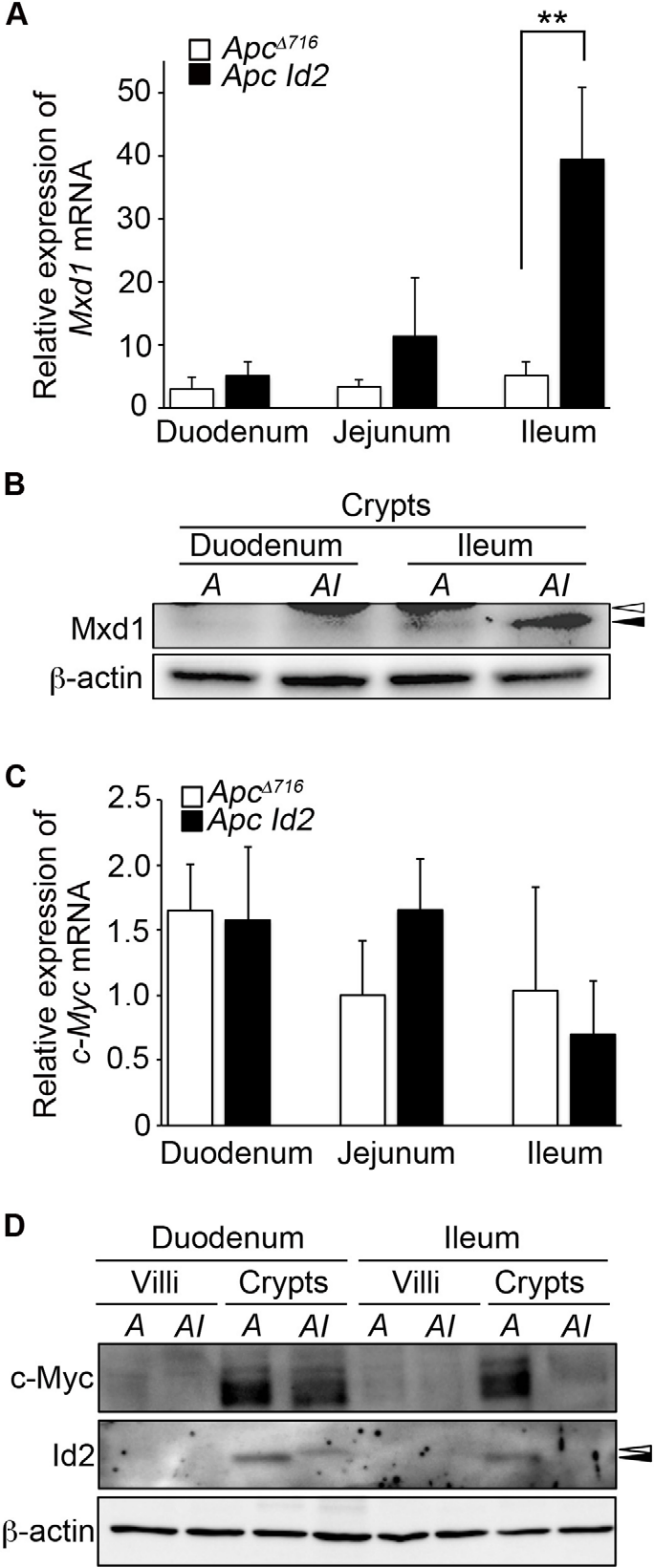
**Loss of Id2 reduces the protein level of c-Myc and increases the mRNA level of its antagonist Mxd1 in the ileal crypts of *Apc*^Δ716^ mice.** (A) Expression of *Mxd1* mRNA determined by qRT-PCR. Total RNA was prepared from isolated crypts in the normal duodenum, jejunum and ileum of *Apc*^Δ716^ and *Apc Id2* mice. The results are given as means±s.d. (*n*=5). **P<0.01. (B) Expression of Mxd1 protein determined by western blotting. Protein samples were prepared from isolated crypts in the normal duodenum and ileum of *Apc*^Δ716^ and *Apc Id2* mice, as shown in Fig. 3A. The open and closed arrowheads indicate nonspecific bands and specific Mxd1 bands. *A*, *Apc*^Δ716^ mouse; *AI*, *Apc Id2* mouse. β-actin, loading control. (C) Expression of *c-Myc* mRNA determined by qRT-PCR. Total RNA was prepared from isolated crypts in the normal duodenum, jejunum and ileum of *Apc*^Δ716^ and *Apc Id2* mice. The results are given as means±s.d. (*n*=5). (D) Expression of c-Myc protein determined by western blotting. Protein samples were prepared from isolated crypts, as shown in Fig. 3A. The open and closed arrowheads indicate a nonspecific band and specific Id2 bands, respectively. *A*, *Apc*^Δ716^ mouse; *AI*, *Apc Id2* mouse. β-actin, loading control.

To investigate the genes that could be involved in the inhibition of ileal tumor formation in *Apc Id2* mice, we next performed DNA microarray analysis of ileal crypts that were isolated from *Apc*^Δ716^ and *Apc Id2* mice. We then applied the following criteria to select for such genes. First, the expression of the candidate gene was up- or down-regulated by more than 8-fold in the microarray analysis. Second, the expression levels of the candidate genes are changed in the ileal crypt, yet not in the duodenum or jejunum. Third, the candidate-genes have been implicated in tumor initiation. Although 183 and 57 genes were up- and down-regulated, respectively, by the Id2 loss (supplementary material Table S2), *Max dimerization protein 1* (*Mxd1*) was the only candidate gene that met the three criteria. It is known that Mxd1 antagonizes c-Myc function by competing with c-Myc for interaction with Max (Ayer et al., 1993). It has been also reported that c-Myc is one of the most important downstream targets of Wnt signaling (He et al., 1998) and functions as a mediator of polyp growth and intestinal cancer initiation (Ignatenko et al., 2006; Sansom et al., 2007; Yekkala and Baudino, 2007). As shown in Fig. 5A, loss of *Id2* increased the mRNA level of *Mxd1* by ∼8 fold in the crypt epithelium of the ileum but not in that of the duodenum or jejunum. Induction of Mxd1 expression by Id2 deletion was also observed at protein level, as shown by western blot (Fig. 5B). To analyze the functional interaction between c-Myc and Mxd1, we next determined the expression levels of c-Myc in the intestinal crypt. Lack of Id2 did not affect the level of *c-Myc* mRNA throughout the small intestinal tract (Fig. 5C). Importantly, loss of Id2 down-regulated the protein level of c-Myc in the ileum but not in the duodenum of *Apc*^Δ716^ mice (Fig. 5D). According to these results, Id2 ablation also affects translation or stabilization of c-Myc protein as well as transcription of *Mxd1* mRNA. These results suggest that loss of Id2 inhibits tumor initiation through up-regulation of *Mxd1* mRNA and down-regulation of c-Myc protein.

## DISCUSSION

We have shown here direct genetic evidence that Id2 plays a crucial role in ileal tumor initiation in *Apc*^Δ716^ mice. Although Apc-deficient mice have been widely analyzed, this study is the first report to show ileum-specific reduction of tumor multiplicity in these mice.

Id2 is highly expressed in the intestinal crypt epithelium and its expression level is further increased in Apc-deficient adenoma (Figs 1 and 3). It is known that Wnt signaling is activated in the intestinal crypt and adenoma epithelial cells (Reya and Clevers, 2005). Consistent with these findings, our results have shown that Tcf4 binds directly to the enhancer region of the *Id2*-gene promoter (Fig. 1D). In addition, the encyclopedia of DNA elements (ENCODE) data also showed that TCF4 binds to a region of the human *ID2* promoter that is homologous to the mouse *Id2* enhancer in HCT116, a Wnt-signaling activated colon cancer cell line (Birney et al., 2007) (supplementary material Fig. S1). These findings imply that the expression of *ID2/Id2* is stimulated as a downstream target gene of Wnt signaling in the intestinal crypts and tumor epithelium of both humans and mice.

Earlier studies have shown that Id2 contributes to tumor proliferation. Overexpression of Id2 increases anchorage-independent proliferation of both SW480 and HT29 colon cancer cells (Rockman et al., 2001), and knockdown of Id2 decreases cell proliferation of HCT116 cells (Gray et al., 2008). On the other hand, we have demonstrated here that loss of Id2 inhibits tumor initiation rather than tumor expansion in *Apc*^Δ716^ mice (Fig. 2B,C). Of note, adenoma epithelial cells of *Apc*^Δ716^ mice do not carry additional mutations other than *Apc* (Oshima et al., 1995a). In contrast, human colon cancer cell lines that have been used in previous reports have several mutations such as K-ras, p53 and transforming growth factor (TGF)-β type II receptor (Gayet et al., 2001; Ijichi et al., 2001). It is therefore conceivable that Id2 is involved in the proliferation of adenocarcinoma epithelium beyond the stage examined here.

O'Brien et al. have reported that Id1 and Id3, other Id family members, are expressed in colon cancer initiating cells and function together with p21 to regulate their self-renewal capacity through cell cycle restriction and DNA damage accumulation (O'Brien et al., 2012). A more recent study has also shown that loss of Id1 inhibits tumor initiation but does not affect tumor expansion in *Apc^Min/+^* mice (Zhang et al., 2015). These findings and our study show that Id2 plays an important role in intestinal tumor initiation in addition to Id1 and Id3. This raises the possibility that Id1 and Id3 compensate for the function of Id2 in the duodenal- and jejunal-tumor initiation that is not affected by the loss of Id2.

Our results suggest the possibility that the enhanced expression of Mxd1 suppresses ileal-tumor initiation in *Apc Id2* mice (Fig. 5A,B). Mxd1 belongs to the Max dimerization protein (Mxd) family of proteins, which function as potent antagonists of c-Myc. It is currently understood that Mxd1 competes with c-Myc for the transcription factor Max and for their recognition sequence E-box (Cascón and Robledo, 2012; Diolaiti et al., 2015). The Mxd1–Max heterodimer then functions as a transcriptional repressor at E-box containing promoters and antagonizes the c-Myc–Max functions. Consistently, overexpression of Mxd1 inhibits c-Myc/Ha-Ras mediated co-transformation in rat embryo cells (Cerni et al., 1995). In addition, loss of Mxd2, a member of the Mxd family, resulted in susceptibility to tumorigenesis in squamous cell carcinoma of the skin and malignant lymphoma (Schreiber-Agus et al., 1998). It is therefore possible that Mxd1 contributes to the tumor initiation of various tissues including ileum.

c-Myc is a critical mediator of adenoma initiation following *Apc* loss (Ignatenko et al., 2006; Sansom et al., 2007; Yekkala and Baudino, 2007). It was reported that targeted c-Myc deletion in the intestinal epithelium reduced the tumor number of *Apc* heterozygotic (*Apc*^Min/+^) mice with increases in apoptosis (Ignatenko et al., 2006). Consistent with those studies, our results show that down-regulation of c-Myc is accompanied by increases in apoptosis in the intestinal crypt epithelium of *Apc Id2* mice (Figs 4A,B and 5B). However, it has also been reported that conditional *Myc* gene ablation decreases the number of apoptotic cells in the intestinal epithelium of *Apc* homozygotic (*AhCre Apc^fl/fl^*) mice (Sansom et al., 2007). This discrepancy in the apoptotic phenotypes may be attributable to the number of *Apc* mutant alleles in the normal intestinal epithelium. Further studies are required to understand the precise roles of Id2, c-Myc and Apc in apoptosis of intestinal epithelial cells.

Curiously, Id2 deletion decreased c-Myc protein levels, but not mRNA levels, in the small-intestinal ileal crypt (Fig. 5C,D). Importantly, it is known that Mxd1 can inhibit c-Myc function by prevention of c-Myc–Max heterodimerization (Ayer et al., 1993). This raises a possibility that inhibition of c-Myc–Max heterodimer formation by enhanced expression of Mxd1 is involved in destabilizing c-Myc protein. Thus, it is likely that Id2 promotes tumor initiation through inhibition of the Mxd1–Max network and activation of the c-Myc–Max.

In conclusion, we have demonstrated that Id2 is transcriptionally controlled by Wnt signaling and contributes to the cell survival of distal-intestinal crypt epithelium, which is accompanied by the suppression of Mxd1 and stabilization of c-Myc.

## MATERIALS AND METHODS

### Mice

The knockout alleles of the *Apc* and *Id2* genes have been described previously (Oshima et al., 1995a; Yokota et al., 1999). The *Apc*^Δ716^ allele on the C57BL/6 background was transferred to the 129/Sv genetic background by backcrossing for more than 5 generations. Female *Apc*^Δ716^ mice on the 129/Sv background were then crossed twice with the *Id2*^−/−^ mice on the 129/Sv background to generate double mutant Apc *Id2*^−/−^ mice. All animal experiments were carried out according to the protocols approved by the Animal Care and Use committee of University of Fukui.

### Cell culture

HCT116 and SW480 cells were obtained from the American Type Culture Collection. The cells were grown at 37°C in 5% CO_2_ using DMEM (Wako, Osaka, Japan) supplemented with 10% fetal bovine serum (Thermo Fisher Scientifics, Waltham, MA, USA) and penicillin/streptomycin (Wako).

### RT-PCR

The levels of specific mRNAs were determined by quantitative reverse-transcription polymerase chain reaction (qRT-PCR) using a StepOnePlus Real Time PCR system (Life Technologies, Foster, CA, USA). This analysis was adapted from the previous study (Kakizaki et al., 2010). Primers used were as follows: mouse *Actb* (forward, 5′-TGACAGGATGCAGAAGGAGA-3′; reverse, 5′-GCTGGAAGGTGGACAGTGAG-3′), mouse *Id2* (forward, 5′-ACTCGCATCCCACTATCGTCAG-3′; reverse, 5′-TGCTATCATTCGACATAAGCTCAGA-3′), mouse *Id1* (forward, 5′-GGAGCTGAACTCGGAGTCTGAA-3′; reverse, 5′-GATCGTCGGCTGGAACACA-3′), mouse *Id3* (forward, 5′-GGAAATCCTGCAGCGTGTCATA-3′; reverse, 5′-GAGATCACAAGTTCCGGAGTGA-3′), mouse *c-Myc* (forward, 5′-CGAGCTGTTTGAAGGCTGGA-3′; reverse, 5′-GTCGCAGATGAAATAGGGCTGT-3′), mouse *Mxd1* (forward, 5′-GACAGCGTGGGCTCTGTG-3′; reverse, 5′-CTGTGCCCTCCACATCCAC-3′), mouse *Cdx1* (forward, 5′-ACAGCCGGTACATCACTATCC-3′; reverse, 5′-GTTTACTTTGCGCTCCTTGG-3′), mouse *Cdx2* (forward, 5′-TCTCCGAGAGGCAGGTTAAA-3′; reverse, 5′-CTGTGGAGGCTGTTGTTGCT-3′), and mouse *Gata4* (forward, 5′-CCGGGCTGTCATCTCACTAT-3′; reverse, 5′-CAGACAGCACTGGATGGATG-3′).

### Laser microdissection

The mouse small intestinal epithelium was captured from frozen sections using an AS LMD (Leica Microsystems, Bannockburn, IL, USA), and the RNAs were purified using an RNeasy Micro Kit (Qiagen, Hilden, Germany).

### Western blotting

Dissected tissue samples were homogenized in RIPA lysis buffer containing 50 mM Tris-HCl, pH 7.4; 150 mM NaCl; 1 mM NaF; 1% NP-40; 0.25% sodium deoxycholate; 1 mM sodium orthovanadate; 1 mM EDTA and a protein protease inhibitor cocktail (Nacalai Tesque, Kyoto, Japan). Lysates containing 10–40 µg of protein were separated by SDS-PAGE and transferred to Hybond ECL Nitrocellulose membranes (GE Healthcare, Buckinghamshire, UK). The membranes were blocked with Blocking One (Nacalai Tesque) for 1 h at room temperature. The membranes were then probed with the following antibodies: rabbit polyclonal anti-Id2 (sc-489; Santa Cruz Biotechnology, Dallas, TX, USA), rabbit monoclonal anti-Id2 (D39E8, #3431; Cell Signaling Technology, Beverly, MA, USA), rabbit polyclonal anti-Mxd1/Mad1 (sc-222; Santa Cruz Biotechnology), rabbit monoclonal anti-c-Myc (D84C12, #5605; Cell Signaling Technology) and mouse monoclonal anti-β-actin (Sigma-Aldrich, Saint Louis, MO, USA) antibodies. After incubation with horseradish peroxidase-conjugated secondary antibodies, bound proteins were detected by incubation with an Immobilon chemiluminescent substrate (Millipore, Billerica, MA, USA).

### Polyp number scoring

Dissected intestinal tissues were fixed with 4% paraformaldehyde. The number of polyps was scored as described previously (Oshima et al., 1995a).

### Histological analysis

Dissected intestinal tissues were fixed with 4% paraformaldehyde, embedded in paraffin wax and sectioned at a thickness of 4 µm. These sections were stained with H&E or were processed for further immunostaining as described previously (Oshima et al., 1995b). Rabbit polyclonal anti-cleaved caspase3 (Asp175, #9661; Cell Signaling Technology), anti-Ki67 (RM-9106; Thermo Fisher Scientifics) were used as primary antibodies.

### Classification of polyps

Dissected intestinal tissues were fixed with 4% paraformaldehyde, embedded in paraffin wax and sectioned at a thickness of 4 µm at 40 µm intervals throughout the tissue block and stained with anti-β-catenin (c2206; Sigma-Aldrich) antibodies. Polyps were categorized as β-catenin-positive or β-catenin-negative and scored for numbers.

### DNA microarray

Normal ileal crypts were isolated from the *Apc*^Δ716^ and *Apc Id2* mice as previously described (Sato et al., 2009). Total RNA was purified using the RNeasy Micro Kit (Qiagen). Gene expression profiles were analyzed using the 3D-Gene Mouse Oligo Chip 24k (Toray Industries, Kanagawa, Japan).

### ChIP-PCR

ChIP assays were performed using the ChIP-it high sensitivity kit (Active Motif, Carlsbad, CA, USA) according to the manufacturer's instructions. Intestinal polyps from *Apc*^Δ716^ mice were fixed with 1% formaldehyde for 15 min at room temperature. Isolated nuclei from the mouse intestinal polyps were sonicated using a Biorupter (Cosmo Bio Co. Ltd., Tokyo, Japan). After centrifugation to remove debris, chromatin samples were incubated with rabbit monoclonal anti-TCF4 antibody (C48H11, #2569; Cell Signaling Technology) or normal rabbit IgG (#2729; Cell Signaling Technology) overnight at 4°C. The qPCR analysis of immunoprecipitated DNA fragments was performed using KOD SYBER qPCR Mix (TOYOBO, Osaka, Japan) with the following primer set: mouse *Axin2* promoter (forward, 5′-CTCGCATACCTCCCTTCC-3′; reverse 5′-TTCCAGCAGTCACTAGGC-3′), mouse *Id2* promoter-1 (forward, 5′-GGGTGCTGAAAGATTCCAAA-3′; reverse 5′-GTGGCAAAGTGATGCGATTA-3′), mouse *Id2* promoter-2 (forward, 5′-TCAGTACCGCAGAGATGCAA-3′; reverse 5′-GCGGAACAGATTTTGTTTTGA), or mouse *Id2* promoter-3 (forward, 5′-TTTTGGAGTCAAAGCCATCC-3′; reverse 5′-AGACCTTGCCAAAGCAAAAG-3′). Results are presented as percentage immunoprecipitated over input and are representative of three independent experiments.

### Vector construction and site-directed mutagenesis

The full length, *TCF4*/*TCF7L2* cDNAs were isolated by PCR-based cloning techniques using the following primer sets: *TCF4*/*TCF7L2* (forward, 5′-CTTCCTCCTTCATTTTTCTT-3′; reverse, 5′-CAATGAACTCGATAAACATC-3′). The *TCF4*/*TCF7L2* cDNAs was inserted into pcDNA6/V5-HisA (Invitrogen, Grand Island, NY, USA) for transient expression. The putative *Id2* enhancer region was cloned by PCR amplification from the genomic DNA from mouse tail using the forward primer 5′-GCGTTTCCAGGCTGACTTAC-3′ and the reverse primers 5′-AGACCTTGCCAAAGCAAAAG-3′, and inserted into pGL4.10 luciferase vector (Promega, Madison, WI, USA) to generate the reporter construct (Fig. 1C). The pCMV6-XL5 expression vector carrying the *CTNNB1* was obtained from T. Akiyama (The University of Tokyo). Site-directed mutagenesis for mutating the *CTNNB1* was performed using PrimeSTAR mutagenesis Basal kit (TaKaRa, Shiga, Japan) with the following oligomers: 5′-CAGTCTTACCTGGACT**a**TGGAATCCATTCTGGTG-3′ and 5′-CACCAGAATGGATTCCA**t**AGTCCAGGTAAGACTG-3′. The lower cases indicate the mutated sequences in the CTNNB1 phosphorylation sites. The mutated cDNA were subcloned into pcDNA6/V5-HisA.

### Luciferase reporter gene assay

The luciferase assay protocol was adapted from a previous study (Kakizaki et al., 2010). HCT116 or SW480 cells were seeded into MP-24 tissue culture plates at a density of 5×10^4^ cells per well. The cultures were transfected with 100 ng of luciferase reporter gene constructs, 200 ng of a pcDNA6/V5-HisA expression vector carrying the *CTNNB1* S33A mutant encoding the active form of β-catenin and *TCF4*, and 2 ng of the hRluc-CMV vector (Promega), using LipofectaminLTX (Life Technologies). After 24 h of incubation, the cultures were washed once with ice-cold PBS and then lysed in 100 µl of Passive lysis buffer (Promega) according to the manufacturer's protocol. Firefly and Renilla luciferase activities were determined using the Dual-Luciferase Reporter Assay System (Promega) with a MITHORAS LB 940 (Berthold Biolumat, Bad Wildbad, Germany). Firefly luciferase activities were normalized against the Renilla luciferase activities.

### Statistical analysis

The values are shown as means±s.d. Data were analyzed using a Student's *t*-test or ANOVA. *P*-values smaller than 0.05 were considered statistically significant.

### Accession numbers

Microarray hybridization data have been deposited in the GEO database with accession code GSE66480.

## Supplementary Material

Supplementary Material
