# Supplementary material for: Id2 deletion attenuates Apc-deficient ileal tumor formation
Source: Biol Open. 2015 Jul 10;4(8):993–1001. doi: 10.1242/bio.012252 (PMC4542283; doi:10.1242/bio.012252)
Supplement: Supplementary Material [file supp_4_8_993__index.html]

Id2 deletion attenuates Apc-deficient ileal tumor formation — Id2 deletion attenuates Apc-deficient ileal tumor formation — Supplementary Material 

# Id2 deletion attenuates *Apc*-deficient ileal tumor formation

## BIO012252 Supplementary Material

- Supplementary Material
