# Supplementary material for: Id2 deletion attenuates Apc-deficient ileal tumor formation
Source: Biol Open. 2015 Jul 10;4(8):993–1001. doi: 10.1242/bio.012252 (PMC4542283; doi:10.1242/bio.012252)
Supplement: Supplementary Material [file supp_bio.012252_BIO012252supp.pdf]

Supplementary Material

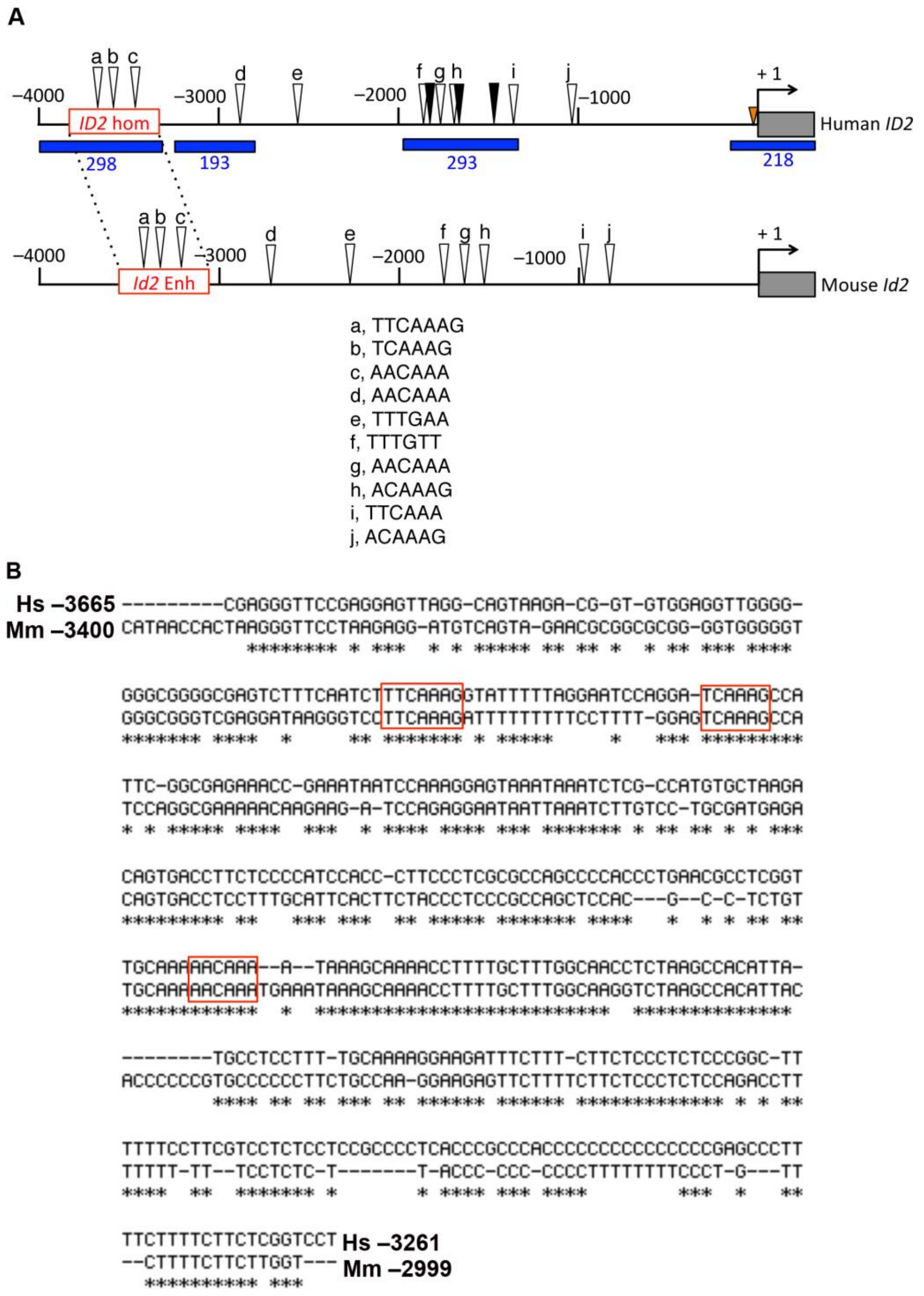

Fig. S1. TCF/LEF recognition sequences are conserved between human and mouse *ID2/Id2* promoters. (A) Schematic representation of TCF/LEF binding sequences of the human *ID2* and mouse *Id2* promoter regions. The gray rectangles indicate the first

exons of human *ID2* and mouse *Id2*. The numbers indicate the distance from the *ID2*/*Id2* transcriptional start sites. The open and filled arrowheads indicate conserved and non-conserved TCF/LEF binding consensus sequences, respectively. The orange arrowhead indicates the TCF/LEF binding site reported previously (Rockman et al., 2001). Blue bars and numbers indicate the TCF4-binding regions and -affinity indexes reported by the ENCODE consortium (Birney et al., 2007). a-j, conserved TCF/LEF recognition sequences between humans and mice which are indicated by open arrowheads in the upper panel. (B) Alignment between the human *ID2* and mouse *Id2* promoter sequences. Numbers indicate the distance from the *ID2/Id2* transcription start sites. TCF/LEF-binding consensus sequences are indicated by red solid boxes.

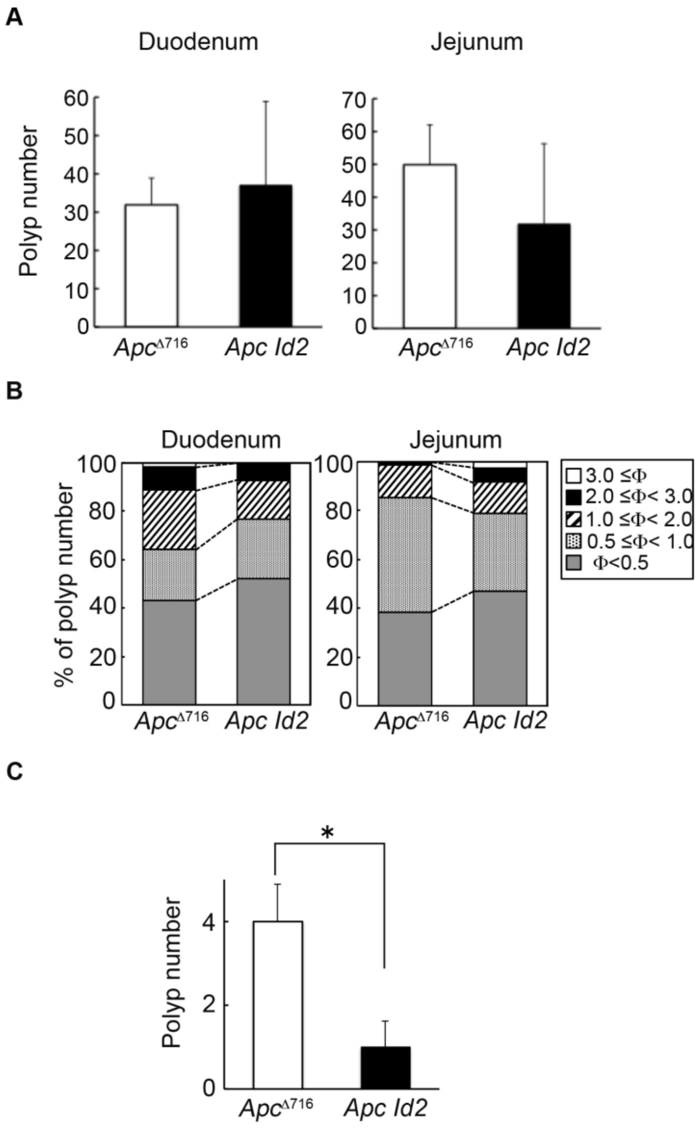

Fig. S2. Id2-deficiency suppresses tumor initiation in the ileum but not in the duodenum and the jejunum. (A) The total number of polyps in the duodenum and jejunum of *Apc*$^{\Delta716}$ and *Apc Id2* mice at 16 weeks of age as assessed using a dissecting microscope. The results show the mean number of polyps/mouse±s.d. (*n*=7-8). (B) The size distribution of polyps in the duodenum and jejunum. Polyps were classified according to their diameters (Φ) in millimeters. (C) Total number of ileal polyps in *Apc*$^{\Delta716}$ and *Apc Id2* mice at 4 weeks of age as assessed using a dissecting microscope **P<0.01.

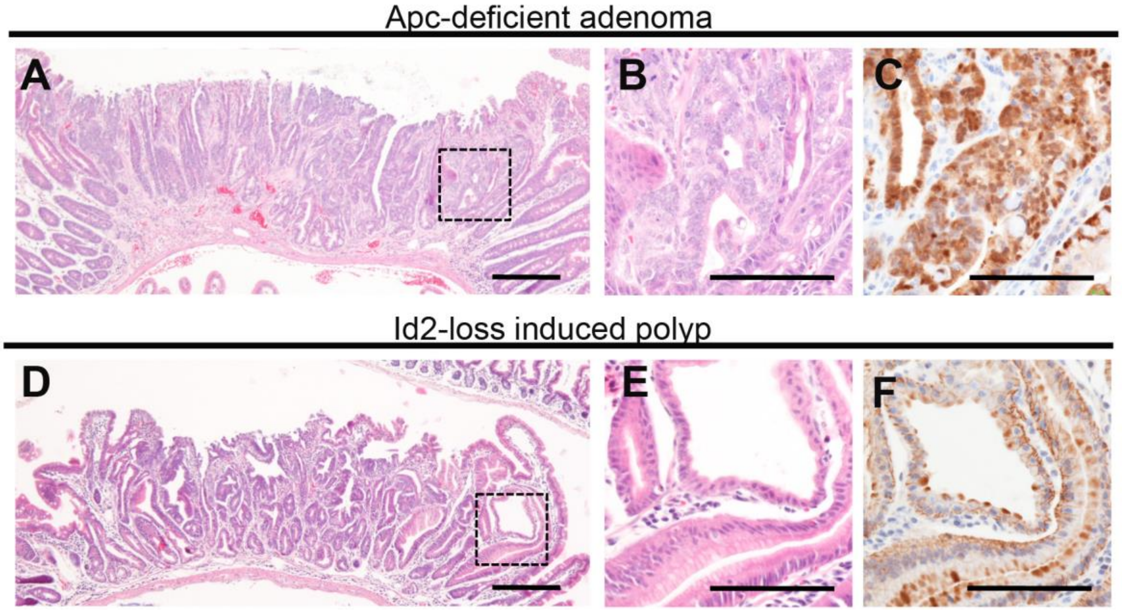

Fig. S3. β-catenin staining of polyps induced by the loss of Id2 or the LOH of *Apc*. Intestinal tumors in *Apc Id2* mice were stained with H&E (A, B, D, and E) or β-catenin (C and F). Scale bars in A and D indicate 200 µm. B and E show higher magnification of the dotted boxes in A and D, respectively. Scale bars in B, C, E, and F indicate 100 µm.

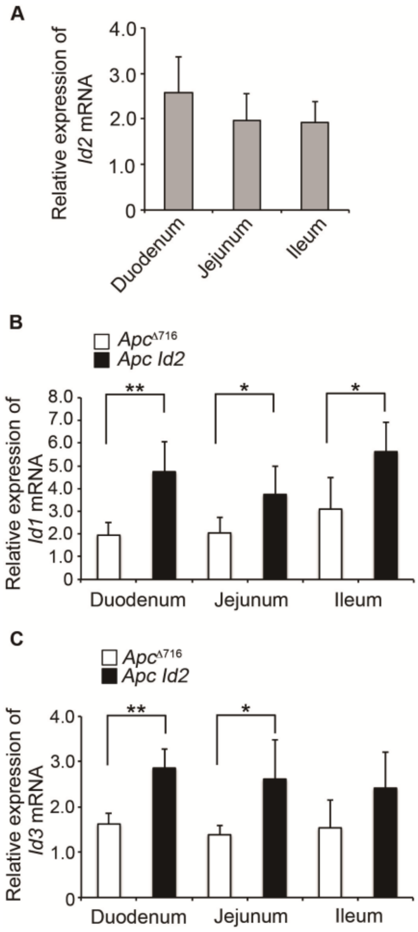

Fig. S4. Expression of Id1, Id2, and Id3 along the proximal and distal axes of small intestinal crypts of *Apc*$^{\Delta716}$ mice. (A) Relative expression of *Id2* mRNA in normal small intestinal crypts in the duodenum, jejunum, and ileum of *Apc*$^{\Delta716}$ mice determined by qRT-PCR. The results are given as means±s.d. (*n*=5). (B-C) Relative expression of *Id1* (B) and *Id3* (C) mRNA in normal small intestinal crypts in the duodenum, jejunum, and ileum of *Apc*$^{\Delta716}$ and *Apc Id2* mice, determined by qRT-PCR. The results are given as means±s.d. (*n*=5). *P < 0.05, **P <0.01. RNA samples were isolated from the normal epithelium of crypts of *Apc*$^{\Delta716}$ or *Apc Id2* mice, as shown in Fig. 3A.

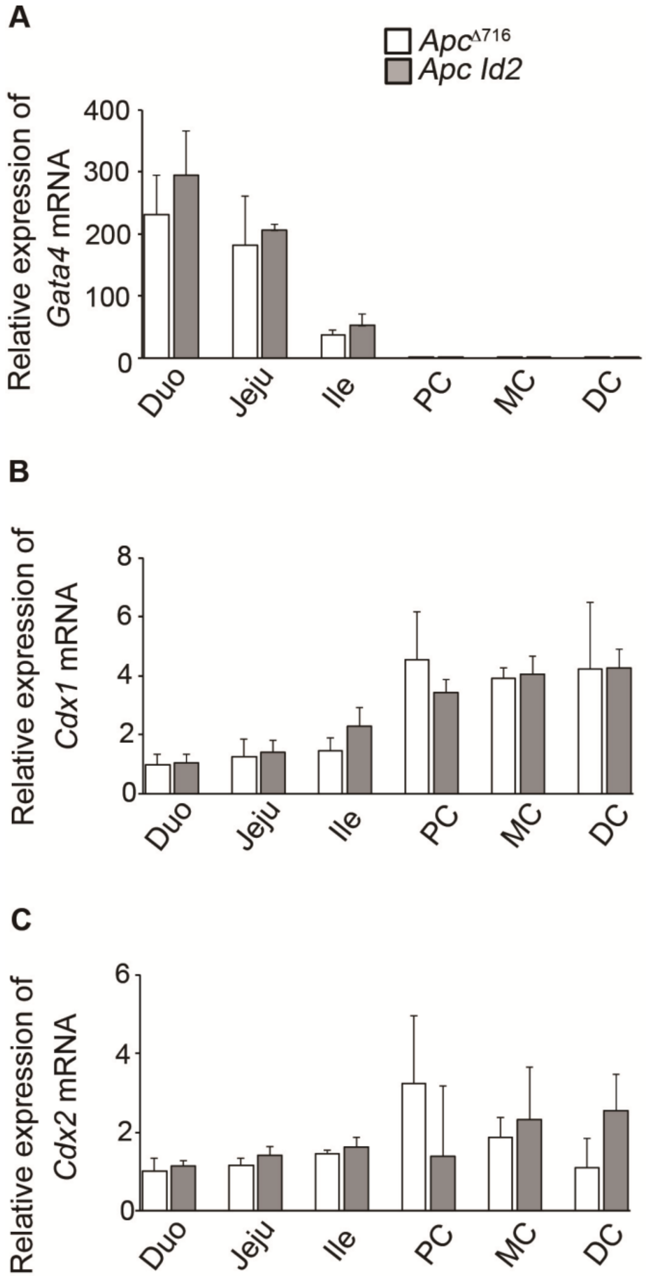

Fig. S5. Effects of loss of Id2 on the expression of genes that define the boundary at jejunum-ileum and/or ileum-colon junction. (A-C) Relative expression of *Gata4* (A), *Cdx1* (B), and *Cdx2* (C) mRNA in the indicated regions of the normal intestine of *Apc*$^{\Delta716}$ and *Apc Id2* mice, determined by qRT-PCR. Duo, duodenum; Jeju, jejunum; Ile, ileum; PC, proximal colon; MC, mid colon; DC, distal colon. The results are given as means±s.d. (*n*=5).

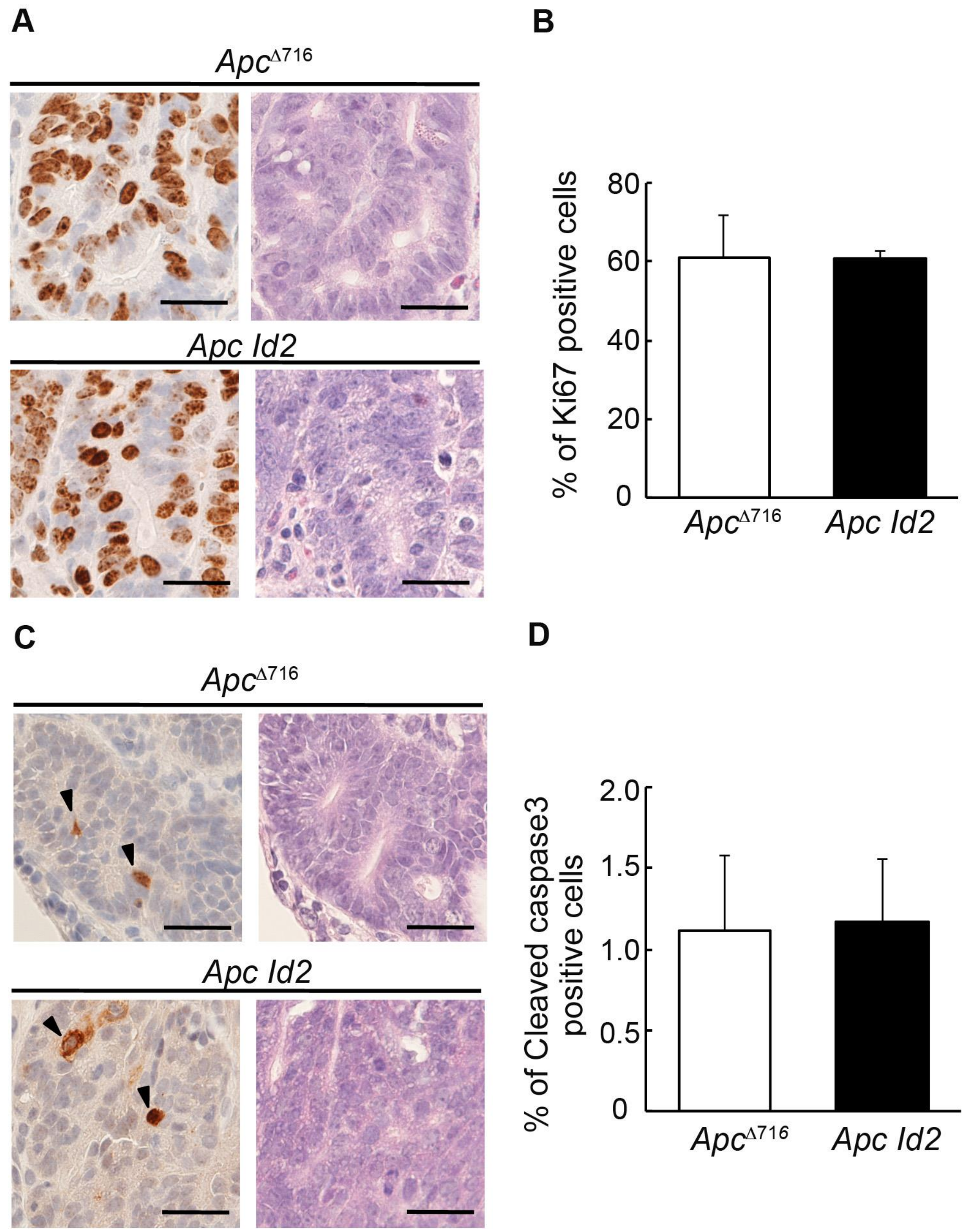

Fig. S6. Effects of Id2-loss on adenoma epithelial cell growth and apoptosis in *Apc*$^{\Delta716}$ mice. (A) Immunohistochemical staining of adenoma cells of the ileal polyps of 16 week-old *Apc*$^{\Delta716}$ (upper) and *Apc Id2* (lower) mice for Ki67 (left panels). Serial sections were stained with H&E (right panels). Scale bars, 50 μm. (B) Ki67 labeling

index of the ileal polyps of *Apc*$^{\Delta716}$ and *Apc Id2* mice. (C) Apoptotic cells in ileal polyps of 16 week-old *Apc*$^{\Delta716}$ (upper) and *Apc Id2* (lower) mice were stained with anti-cleaved caspase-3 antibody (left panels, indicated by arrowheads). Serial sections were stained with H&E (right panels). Scale bars, 50 μm. (D) Apoptotic indices of ileal polyps in the *Apc*$^{\Delta716}$ and *Apc Id2* mice.

Table S1. Number of Id2 loss-induced polyps or Apc-deficient adenomas in the small intestine of *Id2*$^{-/-}$, *Apc*$^{\Delta716}$ and *Apc Id2* mice.

	Id2 loss-induced polyps	Apc-deficient adenomas	Id2 loss-induced polyps (%)
Id2$^{-/-}$	4.7 ± 2.9	—	100
Apc$^{\Delta716}$	—	176.0 ± 22.6	0
Apc Id2	5.3 ± 5.9	145.7 ± 24	4.1 ± 4.7

The results are given as means ± S.D. ($n = 4$).

Table S2

Click here to Download Table S2
